# Depression and Anxiety among Patients with Type II Diabetes Mellitus in Chitwan Medical College Teaching Hospital, Nepal

**DOI:** 10.1155/2021/8846915

**Published:** 2021-01-13

**Authors:** Kalpana Sharma, Govinda Dhungana, Shailendra Adhikari, Archana Bista Pandey, Muna Sharma

**Affiliations:** ^1^School of Nursing, Chitwan Medical College Affiliated to Tribhuvan University, Bharatpur 44207, Nepal; ^2^Department of Statistics, Birendra Multiple Campus, Tribhuvan University, Bharatpur 44207, Nepal; ^3^Department of Psychiatry, Chitwan Medical College Affiliated to Tribhuvan University, Bharatpur 44207, Nepal; ^4^Maharajgunj Nursing Campus, Institute of Medicine, Tribhuvan University, Bharatpur 44600, Nepal

## Abstract

The prevalence of depression and anxiety disorders is common among people with diabetes mellitus. Coexistence of diabetes and depression/anxiety increases the risk of diabetes complications and reduces the overall quality of life. Hence, this study aimed to assess the depression and anxiety among patients with type 2 diabetes mellitus in Chitwan. Descriptive survey was carried out among 296 purposively selected clinically diagnosed type 2 diabetes patients admitted in the Chitwan Medical College Teaching Hospital from 15^th^ June 2018 to 17^th^ September 2019. Patients were interviewed using the Patient Health Questionnaire-9 (PHQ-9) and Generalized Anxiety Disorders-7 (GAD-7). Data were analyzed using descriptive and inferential statistics. Of 296 diabetic patients, 48.6% were 60 years and above, 59.5% female and 61.5% literate; their common occupation was agriculture (38.2%) followed by household work (26.4%). Nearly two-thirds (62.8%) of diabetes patients had other chronic comorbid conditions. Depression and anxiety were observed among 57.8% and 49.7% of diabetes patients, respectively. While observing the severity, 27.4%, 19.6%, 8.4%, and 2.4% of patients had mild, moderate, moderately severe, and severe depression, respectively. Likewise, 24.7%, 20.3%, and 4.7% of patients had mild, moderate, and severe anxiety, respectively. Current living status, educational status, medicine adherence, satisfaction toward current treatment, and history of mental illness in the family were found to be significant factors associated with the anxiety of patients with diabetes. Further, educational status, smoking habit, satisfaction towards current treatment, and history of diabetes in family were the factors associated with depression. Prevalence of depression and anxiety is high among admitted patients with diabetes mellitus, and many factors are associated with it. Hence, regular screening services are essential along with diabetes management plan for timely identification and treatment of the vulnerable groups in the healthcare centers.

## 1. Introduction

Diabetes mellitus (DM) is a frequently encountered chronic metabolic disease which is characterized by elevated plasma glucose level resulting from inadequate insulin secretion and/or increased insulin resistance. According to the International Diabetes Federation (IDF), there were estimated 463 million adults, 20–79 years, living with diabetes mellitus in 2019 which accounted for 9.3% of the global population and was expected to increase to 10.2% (578 million) by 2030 and 10.9% (700 million) by 2045. Although the proportion of people with type 2 diabetes is increasing in most countries, 79% of adults with diabetes are living in low- and middle-income countries [1] and the expected rise in prevalence will be more aggressive in low-income countries [2]. People with DM have a higher risk of morbidity and mortality as well as increased healthcare cost than the general population. Diabetes caused 4.2 million deaths and at least 10% of global health expenditure in 2019 (USD 760 billion dollars) (IDF, 2019) [1]. In Nepal, DM is emerging as a major health problem and its prevalence accounts for 8.4% among adults. The prevalence of diabetes is reported to be higher in urban than in rural areas [3].

Diabetes is typically a manageable disease through lifestyle modifications and treatment. However, it can create added stress to the diabetes patients due to the never-ending demands of diabetes care, such as eating and maintaining physical health, exercising, monitoring blood glucose, regular follow-up, and management of symptoms and fears about or the reality of complications. As a result, they experience feelings of depression, anxiety, and stress, which affect their health and overall quality of life [4].

During psychological stress, counter-regulatory hormones such as catecholamine, a neurotransmitter, glucocorticoids, growth hormones, and glucagon are activated [5]. The activation of the counter regulatory hormones interferes in the action of insulin which is not able to lower glucose but instead elevates blood glucose. The increase in glucose level creates a greater challenge in maintaining metabolic control. Poor glycemic control and functional impairment due to increasing diabetes complications may cause or worsen depression [6] and anxiety in patients [7, 8].

Anxiety and depression are common among patients suffering from type II diabetes, and their prevalence has been summarized in a number of studies [7, 9–11]. Patients with diabetes had significantly higher anxiety and depression than general population [12, 13]. The coexistence of diabetes and depression results in poor glycemic control and self-management, increases the risk of diabetes complications, and reduces overall quality of life and life expectancy [10, 14–16]. So, prevention, early recognition, and treatment of these conditions are essential for achieving optimal goals in the management and in patients' overall quality of life.

In Nepal, depression and anxiety are usually underdiagnosed and undertreated due to social stigma and discrimination as well as there is a dearth in the literatures related to topic. Few previous studies [9, 17–19] reported depression among patients with type II diabetes mellitus attending outpatients' settings. Hence, this study was undertaken to find out depression and anxiety among admitted patients with type II diabetes mellitus which will be helpful for the healthcare providers to plan collaborative care in healthcare settings.

## 2. Materials and Methods

Descriptive cross-sectional survey design was used, and the study was conducted in the Chitwan Medical College Teaching Hospital (CMC-TH), Bharatpur-10, Chitwan, from 15^th^ June 2018 to 17^th^ September 2019. Population of the study was those clinically diagnosed type II diabetes patients either alone or in combination with other diseases admitted in medicine inpatient departments of CMC-TH. Those patients who were clinically diagnosed with type II diabetes according to International Diabetes Federation (IDF) criteria for at least one year irrespective of their sex were included in the study, whereas patients who were unable to complete the interview due to communication or cognitive difficulties were excluded. Sample size was determined using 40.3% overall prevalence of depression among type 2 diabetes at tertiary care centers of Kathmandu [9] with an allowable error of 6% at 95% confidence interval. The estimated sample size was 296 after adding 15% nonresponse rate. A purposive sampling technique was used to select the desired sample for the study.

Pretested Nepali version structured interview schedule was used to collect the sociodemographic and disease-related information of the patients. The Patient Health Questionnaire-9 (PHQ-9) and Generalized Anxiety Disorders-7 (GAD-7) were used for the screening of depression and anxiety. These two instruments assessed the symptoms experienced by participants during the 2-week period before they take the survey. Each item of GAD-7 and PHQ-9 was rated 0 to 3 scores, where 0-not at all, 1-several days, 2-more than half of the days, and 3-nearly every day, with higher scores indicating patients' increased self-report of anxiety and depression severity. The PHQ-9 questionnaire is a validated questionnaire, found to be useful in screening of patients for psychiatric illness worldwide [20–25]. Content and face validity was established through extensive literature review and consultation with experts. Pretesting of the Nepali version instrument was done among 50 diabetes patients admitted in the CMC-TH, and they were excluded from the final study. Reliability of the instrument was tested by calculating Cronbach's alpha values of PHQ-9 and GAD-7 which were 0.78 and 0.75, respectively.

Data were collected through the face-to-face interview method on the 2^nd^ day of admission in a separate room. Scores obtained in GAD-7 were classified into mild (5–9), moderate (10–14), and severe anxiety (≥15). Likewise, scores of PHQ-9 were divided into mild (5 to <10), moderate (10 to <15), moderately severe (15 to <20), and severe (≥20) depression. Those patients who had moderate-to-severe anxiety and depression were referred to the psychiatry department for further management.

To maintain the rights of patients, further evaluation was performed by psychiatrists to those patients who were found to be positive on PHQ-9 and GAD-7 for confirmation and further treatment. Ethical approval was obtained from the Chitwan Medical College Institutional Review Committee (CMC-IRC). Written informed consent was obtained from the patients ensuring their confidentiality of the information.

Collected data were entered into IBM SPSS (Statistical Package for Social Sciences) version 20. Then, data were analyzed in terms of descriptive statistics as well as bivariate analysis. Statistical significance was determined at *p* < 0.05. Then, a multivariate logistic regression model was designed for those variables significant at the bivariate level.

## 3. Results

Of 296 patients, nearly half (48.6%) were 60 years and above and more than half (59.5%) were female. Majority (84.8%) of the patients followed Hinduism, 68.6% were urban residents, 62.5% belonged to joint family, and nearly two-thirds (61.5%) were literate. Common occupation was agriculture (38.2%) and household work (26.4%). More than two-thirds (68.2%) of the patients reported that they quit their job due to their illness. Approximately half (49.7%) of the patients reported that their monthly family income was just sufficient for their daily expenses and 25.3% reported surplus expenses (Table 1).

Regarding clinical variables, more than one-third (37.2%) of the patients had diabetes only, whereas 62.8% had diabetes with other comorbidities. More than three-fourths (76.4%) of the patients' duration of diagnosis was ≥3 years, 53.7% had no history of hospital admission in the last one year, 86.8% had adherence with their medicine, 87.2% were satisfied with their treatment, and only 9.5% had a family history of mental illness (Table 2).

In diabetes patients, overall depression was seen among 57.8% of patients. Among them, 27.4% had mild depression, 19.6% had moderate depression, 8.4% had moderately severe depression, and 2.4% had severe depression. Likewise, overall, 49.7% of patients had anxiety disorder where 24.7%, 20.3%, and 4.7% of patients had mild, moderate, and severe anxiety, respectively (Table 3).

Table 4 shows that the level of anxiety disorder was significantly associated with the sociodemographic variables such as current living status (*p*=0.001) and educational status (*p*=0.001) of the diabetic patients. Likewise, level of depression was significantly associated with the age group in years (*p*=0.045), current living status (*p*=0.003), educational status (*p*=<0.001), smoking habit (*p*=0.005), and perceived impact of illness on work (*p*=0.014).

In Table 5, significant association was found between level of anxiety and selected disease-related variables such as medication adherence (*p*=0.003), satisfaction towards current treatment (*p* < 0.001), and history of mental problem in family (*p*=0.001). Likewise, level of depression was significantly associated with satisfaction towards current treatment (*p*=0.013), other comorbidities (*p*=0.037), history of diabetes in family (*p*=<0.001), and history of mental problem in the family (*p*=0.019) (Table 5).

Logistic regression analysis showed that patients who were currently living with the family, were illiterate, had nonadherence to medication, had a family history of mental diseases, and were not satisfied with current treatment were more likely to be affected by anxiety compared to patients who lived alone, were literate, had adherence to medication, had no history of mental diseases in family, and were satisfied towards current treatment (Table 6).

Regarding depression, patients who were not satisfied towards current treatment, were illiterate, were smokers, and had a family history of diabetes were more likely to have depression than patients who were satisfied with treatment, were illiterate, were never smokers, and had no history of diabetes in the family (Table 7).

## 4. Discussion

This study assessed the depression and anxiety among patients with type 2 diabetes mellitus admitted in a tertiary care center of Chitwan. Of 296 patients with type 2 diabetes mellitus, more than half (57.8%) of the patients exhibited depression and nearly half (49.7%) showed generalized anxiety disorders. Many variables are associated with the level of depression and anxiety of the patients.

The prevalence of depression in our sample is almost similar to the studies conducted among T2DM patients in Kathmandu, Nepal, i.e., 40.3% [9] and 44.1% [19] but higher than the prevalence reported among the patients in Sunsari district (22.7%) [17]. Regarding severity of depression, 27.4%, 14.6%, 8.4%, and 2.4% of patients had mild, moderate, moderately severe, and severe depression, respectively. Although data related to severity from Nepal are limited, a study in Saudi Arabia showed mild, moderate, severe, and extremely severe depression among 9.3%, 14.0%, 7.1%, and 3.3% of patients with type 2 DM, respectively [10].

Compared to other published studies, our finding is almost similar to the study in Pakistan which revealed 49.2% depression in patients with DM [8]. However, prevalence of depression is higher in our sample than that in the studies conducted in Jordan [25], South London King's College hospital [26], Palestine [27], Saudi Arabia [28], and North-Eastcoast Malaysia [29]. Similarly, a meta-analysis of 42 published studies held in the United States reported the prevalence of major depression among 11% of diabetic patients and the prevalence of clinically serious depression in 31% of patients [30]. Likewise, a study carried out in Jordan revealed that the prevalence rate of undiagnosed depression among Jordanian diabetic patients was 19.7% [31]. The possible reasons for the varied prevalence rate of depression in studies might be the use of different scales to screen the depressive symptoms in patients and settings used in these studies.

Along with depression, anxiety is also common among diabetes patients and many studies have reported about it. In our study, half (49.7%) of the patients had anxiety disorders where mild, moderate, and severe anxiety was found in 24.7%, 20.3%, and 4.7% of patients, respectively. Similar results were also reported by studies conducted in Pakistan [8], Saudi Arabia [10, 28], South London King's College hospital [26], and Jordan [25], which found 50.7%, 43.4%, 42.0%, 38.3%, and 37.7% anxiety, respectively, in patients with DM. Our finding is slightly higher than the finding reported by the study in India which showed overall prevalence of anxiety among 34% of patients where mild, moderate, and severe anxiety was found in 22%, 8%, and 4%, of patients, respectively, by GAD-7 scale [24]. In the same line, a study in Saudi Arabia showed mild, moderate, severe and extremely severe anxiety among 13.4, 13.0%, 6.0%, and 5.8% of patients with type 2 DM, respectively [10]. However, very low prevalence of anxiety was observed in other studies conducted in 15 nations [32] and in United States, Baltimore [33] which showed 18.0% and 21.8% overall prevalence of anxiety respectively among type 2 diabetes patients. The variation in the results might be due to nature of the patients included in these studies and different measurement tools.

Bivariate analysis of this study found the significant association of depression with other selected variables such as age in year, current living status, educational status, impact of illness on work, smoking habit, satisfaction towards current treatment, presence of comorbidities, history of diabetes in family, and history of mental problem in family. Regression analysis also found educational status, smoking status, satisfaction toward current treatment, and history of diabetes in family as significant factors associated with depression in the model. These findings are in line with other studies that highlighted the association of depression with patients' educational status, smoking status, family history of diabetes, and compliance with diabetes management [9, 17, 19, 25]. In our study, age and presence of other chronic comorbidities were not significant to the regression model, whereas Ahmad and colleagues reported the significant association of age and presence of ≥ three comorbid diseases than their counterparts in Jordan [25].

Regarding anxiety, our study found the significant association between anxiety and other variables such as current living status, educational status, satisfaction towards current treatment, medicine adherence, and history of mental problem in family. Furthermore, these variables were identified as significant factors associated with anxiety in the regression analysis model. Similarly, another study also showed the consistent results where anxiety was associated with family history of chronic diseases and compliance with diabetes management [10]. However, Ahmad et al. revealed contrasting results where educational level was not significantly associated with the anxiety of patients [25]. In our study, comorbidity was not significant in bivariate analysis as well as regression model, whereas other studies reported the positive association between anxiety and presence of comorbid diseases [10, 26]. This difference in results might be due to varied nature of healthcare systems of the countries and nature of patients included in the study.

This study adds to the dearth of information available regarding anxiety and depression among hospitalized patients with type 2 diabetes mellitus in Nepal. Despite this, it has certain limitations: (i) it is a cross-sectional study which could not explore the causal relationship between anxiety and depression with other associated factors; (ii) this study was conducted among diabetes patients who were admitted in tertiary care hospital setting which may itself mean higher anxiety and depression; (iii) it did not exclude the patients with chronic complications which might have influenced the study findings. Considering these limitations, this study suggested that the coexisting anxiety and depression in diabetes patients needs to be screened regularly in the healthcare settings to enhance the efficacy of treatment regimens and reduce an additional burden on diabetes patients.

## 5. Conclusions

Depression and anxiety are high among the admitted patients suffering from type 2 diabetes mellitus. Many factors such as educational status, current living status, smoking status, medication adherence, satisfaction toward current treatment, and history of mental problem in the family are associated with anxiety and depression. Hence, there is a need to develop an integrated care model to manage these morbidities associated with diabetes mellitus.

## 6. Recommendations

Routine screening and counselling by nurses working in medical departments and regular visits by psychiatrists are recommended for the early detection and treatment of anxiety and depression among diabetes patients admitted in healthcare settings. Many factors are associated with anxiety and depression so these factors are needed to be considered while planning and implementing the program for the risk groups.

## Figures and Tables

**Table 1:** Sociodemographic characteristics of patients.

Sociodemographic characteristics	*n* = 296
	Number (%)
Age groups in years	
<40	12 (4.1)
40–60	140 (47.3)
≥60	144 (48.6)
Mean±SD = 59.50 ± 11.72; min. age: 25 years; max. age: 90	

Sex	
Female	178 (59.5)
Male	120 (40.5)

Religion	
Hindu	251 (84.8)
Other than Hindu^©^	45 (15.2)

Caste	
Bramin	99 (33.4)
Chhetri	47 (15.9)
Janajati	117 (39.5)
Dalit and Madhesi	33 (11.1)

Residence	
Rural	93 (31.4)
Urban	203(68.6)

Family type	
Nuclear	111 (37.5)
Joint	185 (62.5)

Marital status	
Unmarried	5 (1.7)
Married (husband/wife together)	243 (82.3)
Widow/widower	46 (15.5)
Divorce and others	2 (0.7)

Current living status	
Alone	37 (12.5)
With family	259 (87.5)

Educational level	
Illiterate	114 (38.5)
Literate	118 (39.9)
Primary	33 (11.1)
Secondary and above	31 (10.5)

Occupation	
Agriculture	113 (38.2)
Household work	78 (26.4)
Service	48 (16.2)
Business	32 (10.8)
Others (daily wages)	25 (8.5)

Family income	
Insufficient	74 (25.0)
Just sufficient	147 (49.7)
Surplus	75 (25.3)

Impact of illness to quit the job	
Yes	94 (31.8)
No	202 (68.2)

^©^Including Buddhists, Christians, Muslims, Kirats, and others.

**Table 2:** Disease-related characteristics of patients.

	*n* = 296
Variables	Number
Presence of comorbidity	
No	110 (37.2)
Yes	186 (62.8)
Number of comorbidities	
None	110 (37.2)
One	105 (35.4)
Two or more	81 (27.4)
Duration of disease diagnosis in years	
<3 years	70 (23.6)
≥3 years	226 (76.4)
Number of hospital admissions in the last 1 year (*n* = 926)	
None	159 (53.7)
<3	91 (30.7)
3-4	46 (15.5)
Medicine adherence	
Yes	257 (86.8)
No	39 (13.2)
Satisfaction toward current treatment	
Yes	258 (87.2)
No	38 (12.8)
H/o diabetes in family	
Yes	125 (42.2)
No	171 (57.8)
H/o mental problem in family	
Yes	28 (9.5)
No	268 (90.5)

**Table 3:** Level of depression and anxiety among patients with diabetes mellitus.

	*n* = 296
Variables	Number (%)
Level of depression	
Mild	81 (27.4)
Moderate	58 (19.6)
Moderately severe	25 (8.4)
Severe	7 (2.4)
Overall depression	171 (57.8)

Level of anxiety	
Mild	73 (24.7)
Moderate	60 (20.3)
Severe	14 (4.7)
Overall anxiety	147 (49.7)

**Table 4:** Association between anxiety and depression with selected sociodemographic and behavioral pattern of patients.

Variables	Anxiety		*χ*2	*p* value	Depression		*χ*2	*n* = 296
	No	Yes			No	Yes		*p* value
Age group in year								
<40	10 (58.8)	7(41.2)			12 (70.6)	5 (29.4)	6.221	0.045
40–60	72 (47.7)	79 (52.3)	1.122	0.571	59 (39.1)	92 (60.9)		
≥60	67 (52.3)	61 (47.7)			54 (42.2)	74 (57.8)		

Sex								
Female	93 (52.8)	83 (47.2)	1.088	0.297	71 (40.3)	105 (59.7)	0.635	0.426
Male	56 (46.7)	64 (53.3)			54 (45.0)	66 (55.0)		

Residence								
Rural	53 (57.0)	40 (43.0)			45 (48.4)	48 (51.6)	2.107	0.147
Urban	96 (47.3)	107 (52.7)	2.400	0.121	80 (39.4)	123 (60.6)		

Family type								
Nuclear	58 (52.3)	53 (47.7)	0.260	0.610	48 (43.2)	63 (56.8)	0.075	0.785
Joint	91 (49.2)	94 (50.8)			77 (41.6)	108 (58.4)		

Current living status								
Single	28 (75.7)	9 (24.3)	10.860	0.001	24 (64.9)	13 (35.1)	8.880	0.003
With family	121 (46.7)	138 (53.3)			101 (39.0)	158 (61.0)		

Educational status								
Illiterate	44 (38.6)	70 (61.4)	10.224	0.001	33 (28.9)	81 (71.1)	13.408	<0.001
Literate	105 (57.7)	77 (42.3)			92 (50.5)	90 (49.5)		

Occupation								
Agriculture	54 (47.8)	59 (52.2)			43 (38.1)	70 (61.9)		
Homemade work	43 (55.1)	35 (44.9)	1.800	0.615	34 (43.6)	44 (56.4)	1.984	0.576
Service	26 (54.2)	22 (45.8)			20 (41.7)	28 (58.3)		
Business and others	26 (45.6)	31 (54.4)			28 (49.1)	29 (50.9)		

Family income								
Insufficient	35 (47.3)	39 (52.7)	2.006	0.367	30 (40.5)	44 (59.5)	1.499	0.473
Just sufficient	80 (54.4)	67 (45.6)			67 (45.6)	80 (54.4)		
Surplus	34 (45.3)	41 (54.7)			28 (37.3)	47 (62.7)		

Impact of illness on work								
Yes	46 (48.9)	48 (51.1)	0.108	0.742	30 (31.9)	64 (68.1)	6.007	0.014
No	103 (51.0)	99 (49.0)			95 (47.0)	107 (53.0)		

Smoking habit								
Smoking	14 (58.3)	10 (41.7)	0.949	0.622	8 (33.3)	16 (66.7)	10.546	0.005
Past smoker	49 (47.6)	54 (52.4)			32 (31.1)	71 (68.9)		
Never	86 (50.9)	83 (49.1)			85 (50.3)	84 (49.7)		

**Table 5:** Association between anxiety and depression with selected disease-related variables of patients.

Variables	Level of anxiety		*χ*2	*p* value	Level of depression		*χ*2	*n* = 296
	No	Yes			No	Yes		*p* value
Duration of disease								
<3 years	34 (48.6)	36 (51.4)	0.114	0.735	36 (51.4)	34 (48.6)	3.180	0.075
≥3 years	115 (50.9)	111 (49.1)			89 (39.4)	137 (60.6)		

Number of hospital admissions								
None	80 (50.3)	79 (49.7)	2.274	0.321	62 (39.0)	97 (61.0)	2.732	0.255
<3	53 (47.3)	59 (52.7)			49 (43.8)	63 (56.2)		
34	16 (64.0)	9 (36.0)			14 (56.0)	11 (44.0)		

Medicine adherence								
Yes	138 (53.7)	119 (46.3)	8.802	0.003	111 (43.2)	146 (56.8)	0.738	0.390
No	11 (28.2)	28 (71.8)			14 (35.9)	25 (64.1)		

Satisfaction toward current treatment								
Yes	140 (54.3)	118 (45.7)	12.389	<0.001	116 (45.0)	142 (55.0)	6.146	0.013
No	9 (23.7)	29 (76.3)			9 (23.7)	29 (76.3)		

Presence of comorbidity								
No	61 (55.5)	49 (44.5)	1.833	0.176	55 (50.0)	55 (50.0)	4.332	0.037
Yes	88 (47.3)	98 (52.7)			70 (37.6)	116 (62.4)		

Number of comorbidities								
None	61(55.5)	49 (44.5)	2.460	0.292	55 (50.0)	55 (50.0)	4.538	0.103
One	47 (44.8)	58 (55.2)			38 (36.2)	67 (63.8)		
Two or more	41 (50.6)	40 (49.4)			32 (39.5)	49 (60.5)		

H/o diabetes in family								
Yes	65 (52.0)	60 (48.0)	0.239	0.625	37 (29.6)	88 (70.4)	14.147	<0.001
No	84 (49.1)	87 (50.9)			88 (51.5)	83 (48.5)		

H/o mental problem in family								
Yes	6 (21.4)	22 (78.6)	10.339	0.001	6 (21.4)	22 (78.6)	5.485	0.019
No	143 (53.4)	125 (46.6)			119 (44.4)	149 (55.6)		

**Table 6:** Factors associated with the anxiety of patients.

Variables	Unadjusted OR	*p*	Adjusted OR	*p*	*n* = 296 95% CI
Educational status (1-illiterate, 0-literate)	2.169	0.002	2.192	0.003	1.318–3.646
Current living status (1-with family, 0-single)	3.548	0.002	3.098	0.008	1.340–7.164
Medication adherence (1-non-adherence, 0-Adherence)	2.952	0.004	2.791	0.010	1.273–6.122
Satisfaction towards current treatment (1-no, 0-yes)	3.823	0.001	3.744	0.002	1.619–8.662
Mental problem in family (1-yes, 0-no)	4.195	0.003	2.895	0.034	1.081–7.755

Dependent variable: anxiety; Nagelkerke *R*^2^ = 0.196. OR: odds ratio.

**Table 7:** Factors associated with the depression of patients.

Variables	Unadjusted OR	*p*	Adjusted OR	*p*	*n* = 296
					95% CI
Educational status (1-illiterate, 0-literate)	2.509	0.001	2.581	0.001	1.487–4.481
Current living status (1-with family, 0-single)	2.888	0.004	1.335	0.502	0.574–3.104
Smoking status (1-smoker, 0-never smoker)	2.201	0.001	2.072	0.007	1.221–3.516
Impact of illness on work (1-yes, 0-no)	1.894	0.015	1.392	0.261	0.782–2.479
Presence of comorbidity (1-yes, 0-no)	1.657	0.038	1.520	0.132	0.881–2.621
Satisfaction towards current treatment (1-no, 0-yes)	2.632	0.016	3.293	0.009	1.352–8.018
History of DM in family (1-yes, 0-no)	2.522	0.006	2.181	0.006	1.253–3.797
H/o mental problem in family (1-yes, 0-no)	2.928	0.024	1.633	0.340	0.593–4.542

Dependent variable: depression; Nagelkerke *R*^2^ = 0.217. OR: odds ratio.

## Data Availability

The datasets used and/or analyzed during the current study are available from the corresponding author upon request.
